# *OsJAZ4* Fine-Tunes Rice Blast Resistance and Yield Traits

**DOI:** 10.3390/plants13030348

**Published:** 2024-01-24

**Authors:** Mingfeng Zhang, Xiao Luo, Wei He, Min Zhang, Zhirong Peng, Huafeng Deng, Junjie Xing

**Affiliations:** 1 Longping Branch, College of Biology, Hunan University, Changsha 410125, China; mingfz@hnu.edu.cn (M.Z.); xiaoluo@hnu.edu.cn (X.L.); zm18569069524@gmail.com (M.Z.); 2 State Key Laboratory of Hybrid Rice, Hunan Hybrid Rice Research Center, Hunan Academy of Agricultural Sciences, Changsha 410125, China; pzr4321@163.com; 3 National Engineering Laboratory for Rice and By-Product Deep Processing, Central South University of Forestry and Technology, Changsha 410004, China; hewei930312@163.com

**Keywords:** JAZ, jasmonic acid, rice, rice blast, gibberellin, plant immunity

## Abstract

JAZ proteins function as transcriptional regulators that form a jasmonic acid–isoleucine (JA-Ile) receptor complex with coronatine insensitive 1 (COI1) and regulate plant growth and development. These proteins also act as key mediators in signal transduction pathways that activate the defense-related genes. Herein, the role of *OsJAZ4* in rice blast resistance, a severe disease, was examined. The mutation of *OsJAZ4* revealed its significance in *Magnaporthe oryzae* (*M. oryzae*) resistance and the seed setting rate in rice. In addition, weaker *M. oryzae*-induced ROS production and expression of the defense genes *OsO4g10010*, *OsWRKY45*, *OsNAC4*, and *OsPR3* was observed in *osjaz4* compared to Nipponbare (NPB); also, the jasmonic acid (JA) and gibberellin4 (GA4) content was significantly lower in *osjaz4* than in NPB. Moreover, *osjaz4* exhibited a phenotype featuring a reduced seed setting rate. These observations highlight the involvement of *OsJAZ4* in the regulation of JA and GA4 content, playing a positive role in regulating the rice blast resistance and seed setting rate.

## 1. Introduction

Rice (*Oryza sativa* L.) is a staple food. The devastation caused by rice blast due to *Magnaporthe oryzae* (*M. oryzae*) results in a 10–35% loss of the annual global rice harvest [1]. Some studies have emphasized the role of jasmonic acid (JA) in the rice defense response [2,3]. Jasmonates (Jas), including jasmonic acid and its derivatives [4], play a central role in plant resilience against biotic and abiotic stresses [5,6], especially in the activation of plant responses to insect attacks and necrotrophic pathogens [7]. JA signaling pathways are consistent across species, such as *Arabidopsis*, rice, and tomato [8,9]. A co-receptor complex formed between coronatine insensitive 1 (COI1) and jasmonate zim domain (JAZ) is the receptor for jasmonic acid–isoleucine (JA-Ile) [10,11,12]. COI1 is the F-box component of the Skp1/Cullin1/F-box protein ubiquitin E3 ligase (SCFCOI1; [13,14]), while JAZ, part of the TIFY superfamily, acts as a transcriptional regulator protein [10,15].

The JAZ protein encompasses three conserved functional domains: NT, TIFY (also termed as ZIM) [16], and Jas (also named CCT_2) [17]. The NT domain mediates interactions with the DELLA protein to inhibit JA signaling [18]. The ZIM domain, consisting of TIF[F/Y] XG, promotes the formation of JAZ dimers and mediates the interactions between JAZs and the NINJA-TPL complex (TPRs; [19]). The Jas domain consists of SLX2FX2KRX2RX5PY that effectuates physical interactions with COI1 and transcription factors (TFs), such as MYB and MYC [20,21,22]. JAZ plays a crucial role in the JA signaling pathway. Low JA-Ile levels promote JAZ binding to TFs, recruiting corepressors, such as NINJA, TPL, HDA6, and HDA19, to suppress JA-responsive gene expression [19,23]. Post-stimuli, JA-Ile levels rise in plants, leading to JAZ degradation mediated by SCFCOI1-dependent ubiquitination [24]. Consequently, JAZ-inhibited TFs are released that in turn activate the expression of downstream genes. On the other hand, *JAZ4* regulates *Arabidopsis* growth and development independently of or in different branches within the JA signaling pathway [25]. In addition, JAZ plays a central role in the crosstalk among the phytohormone signaling cascades of JA, abscisic acid (ABA), ethylene (ET), and gibberellins (GA) in response to stress [26,27].

The OsJAZ family comprises 15 members [13] that are linked to rice growth, development, and stress responses. The comparison of the various phenotypes between 13 OsJAZs via ectopic expression in *Arabidopsis* and 12 AtJAZs overexpression (OE) lines revealed differences in terms of plant growth, development, and immunity. As an example, compared to other *JAZ* OE plants, *OsJAZ11* OE plants exhibited a JA-insensitive phenotype and enhanced resistance to *Pst* DC3000 [28]. *OsJAZ11* OE also promoted primary and seminal root elongation, which enhanced Pi foraging. Additionally, the ectopic expression of OsJAZ6, which interacts with OsJAZ1, alters the JA signaling and spikelet development in rice [29]. *OsJAZ13* modulates the expression of JA/ET response-related genes that in turn regulates growth and activates hypersensitive cell death [30]. Some studies have shown that the OsJAZ family regulates abiotic and biotic stress responses in rice. The OE of *OsJAZ9* increases the tolerance to salt and dehydration stress in rice [13], while *OsJAZ1* regulates drought resistance partially via the ABA and JA pathways [31]. *OsJAZ8* negatively regulates JA-induced resistance to *Xoo* in rice [32]. OsJAZs mainly function by influencing JA signaling or crosstalk with other phytohormones. In terms of the signal conduction mechanism, recent studies have focused on OsJAZ-OsCOI interaction. A total of 15 OsJAZ homogenes and 3 OsCOI homogenes (OsCOI1a, OsCOI1b, and OsCOI2) in rice are composed of 45 putative OsJAZ-OsCOI co-receptor pairs [33]. OsJAZ2 and OsJAZ5 containing divergent Jas motifs physically interact only with OsCOI2, while OsJAZ4 with a canonical Jas motif interacts with all three rice COIs [34]. OsJAZ1 interacts with OsCOI1b, and the altered interactions of OsJAZ1-OsCOI1b regulate JA signaling during flower and root development [35]. Nonetheless, only a few studies have explored the role of OsJAZ in JA signaling-mediated defense in rice. Herein, we focused on *osjaz4* mutants. *OsJAZ4* knockout reduced the content of endogenous JA and GA4 in rice, weakening the crop’s resistance to blast and reducing the seed setting rate, revealing that *OsJAZ4* positively influences yield traits and immunity against *M. oryzae*.

## 2. Results

### 2.1. Conserved Motifs of OsJAZs and Expression Patterns of OsJAZ Genes in Response to M.oryzae Infection

**Figure 1:**
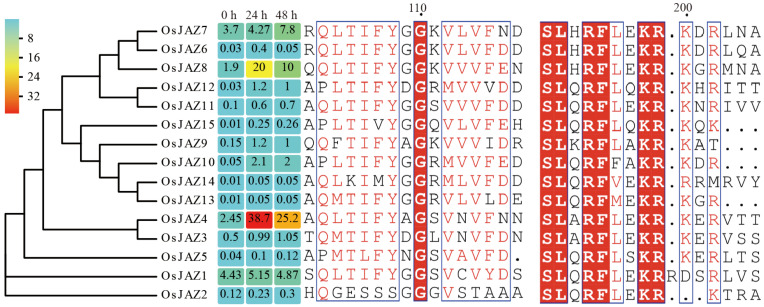
Conserved motifs of OsJAZs and expression patterns of the *OsJAZs* in response to *M. oryzae* infection. Full-length protein sequences of 15 OsJAZs were aligned using ClustalW, and a phylogenetic tree was constructed via the NJ method employing MEGA 11.0.11. The heatmap was constructed using Evolview (https://www.evolgenius.info/evolview) accessed on 26 February 2022 to present the expression patterns of 15 *OsJAZs* genes in the leaves of NPB seedlings inoculated with *M. oryzae* strain 70-15 at 0, 24, and 48 h. The relative expression levels of the target genes were normalized to the level of the *OsActin* (n = 3). The expression levels from low to high are indicated by a change in the color from blue to red. These experiments were performed as independent biological repeats with three technical replicates, each yielding similar results.

For a comprehensive understanding of OsJAZs, the protein sequence was aligned with the conserved sequences using MEGA 11.0.11 (Figure 1). Unlike the *Arabidopsis* JAZ protein, 15 OsJAZs lack an evident NT conservative domain, a weakly conserved region at the N-terminal of the JAZ protein [18]. The conservative domains in OsJAZs are TIFYXG and SLXRFX2KR. Most of the OsJAZ members contain both motifs, with SLXRFX2KR conserved across the family. Notably, OsJAZ2 lacks the TIFYXG domain, OsJAZ14 consists of KIMYXG, and OsJAZ15 harbors TIVYXG, highlighting the conservation of SLXPFX2KR in OsJAZs compared to TIFYXG.

To investigate the reaction of *OsJAZ* genes to rice blast, we exposed NPB seedlings to *M. oryzae* strain 70-15 and examined the expression of *OsJAZs* in leaves via real-time quantitative reverse transcriptase–polymerase chain reaction (qRT-PCR); the relative expression levels were normalized to the level of the *OsActin* (*LOC_Os03g50885*) and the data were visualized as a heatmap (Figure 1). The resulting data consistently showed a similar expression trend when using the rice Ubiquitin gene as the internal reference (Figure S1). The expression of *OsJAZ1*, *OsJAZ7*, and *OsJAZ4* was high in rice seedlings under normal growth conditions (0 h). The relative expression of *OsJAZ4* was raised from 2.45 to 38.7 after 24 h of rice blast infection and 25.2 after 48 h. *OsJAZ10*, *OsJAZ12*, and *OsJAZ15* expression also increased after the rice blast infection, but their expression level was much lower than that of *OsJAZ4*. Also, the expression of *OsJAZ8* was increased. After rice blast infection, the upward adjustment of multiple *OsJAZs* indicated that the *OsJAZ* gene family might have functional redundancy in regulating rice blast resistance. Among these upregulated *OsJAZs*, the expression of *OsJAZ4* was highest in rice leaves. Thus, we speculated a major role of *OsJAZ4* in regulating rice plague resistance, necessitating additional studies on *OsJAZ4*.

### 2.2. Mutation of OsJAZ4 Exacerbates Rice Susceptibility to M. oryzae

**Figure 2:**
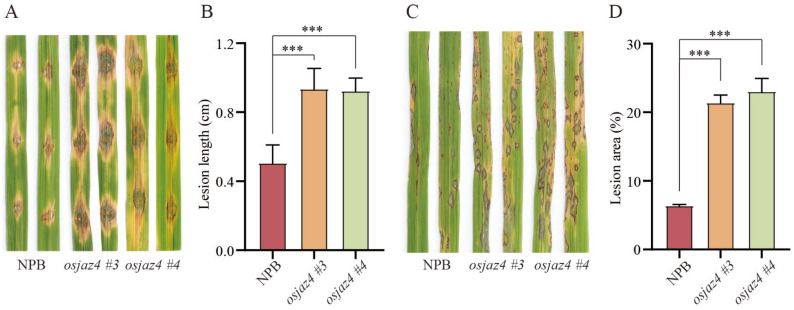
Mutation of *OsJAZ4* exacerbates rice’s susceptibility to *M. oryzae*. (**A**,**B**) Disease symptoms and lesion length of 3-week-old seedlings of NPB; *osjaz4* wound inoculated with *M. oryzae* isolate 70-15 at 6 d post-inoculation (dpi) (n = 15 lesions). (**C**,**D**) Phenotypes of 2-week-old seedlings of NPB; *osjaz4* spray-inoculated with *M. oryzae* isolate 70-15 at 6 dpi (n = 5 leaves). The lesion lengths and percentage of lesion areas were scored via image analysis using ImageJ 1.52i. Error bars indicate standard deviation (SD). Data were statistically analyzed by one-way ANOVA followed by Dunnett’s multiple comparisons test. The asterisks indicate significant differences (*** *p* ≤ 0.001). Three independent biological replicates with three technical repeats were tested, each yielding similar results.

To explore the role of *OsJAZ4* in rice immunity, we generated an *osjaz4* mutant in an NPB background, resulting in two investigation directions: *osjaz4 #3* and *osjaz4 #4* (Figure S2). The leaves of the NPB and *osjaz4* were inoculated with *M. oryzae* strain 70-15 into the wound, and the lesion lengths were assessed on day 6 post-inoculation. The lengths of the lesions induced by 70-15 were notably longer in *osjaz4 #3* and *osjaz4 #4* compared to the control NPB (Figure 2A,B), indicating that the mutation of *OsJAZ4* exacerbates the susceptibility to rice blast fungus. This conclusion was further validated by spray inoculation with 70-15 (Figure 2C,D), wherein the percentage of lesion areas per leaf for *osjaz4* was significantly greater than that of the wild-type (WT) control. These results suggested that *OsJAZ4* enhances rice’s resistance to *M. oryzae*. Furthermore, to clarify whether *OsJAZ4* knockout weakens the broad-spectrum blast resistance of rice, we spray-inoculated NPB and *osjaz4* with three blast isolates (HNB52, HNB119, and Guy11) and found a higher sensitivity of *osjaz4* than NPB in these *M. oryzae* strains (Figure S3). Thus, the *OsJAZ4* gene knockout enhances rice’s susceptibility to *M. oryzae*.

### 2.3. The Immunity Response of osjaz4 against M. oryzae Is Weakened

**Figure 3:**
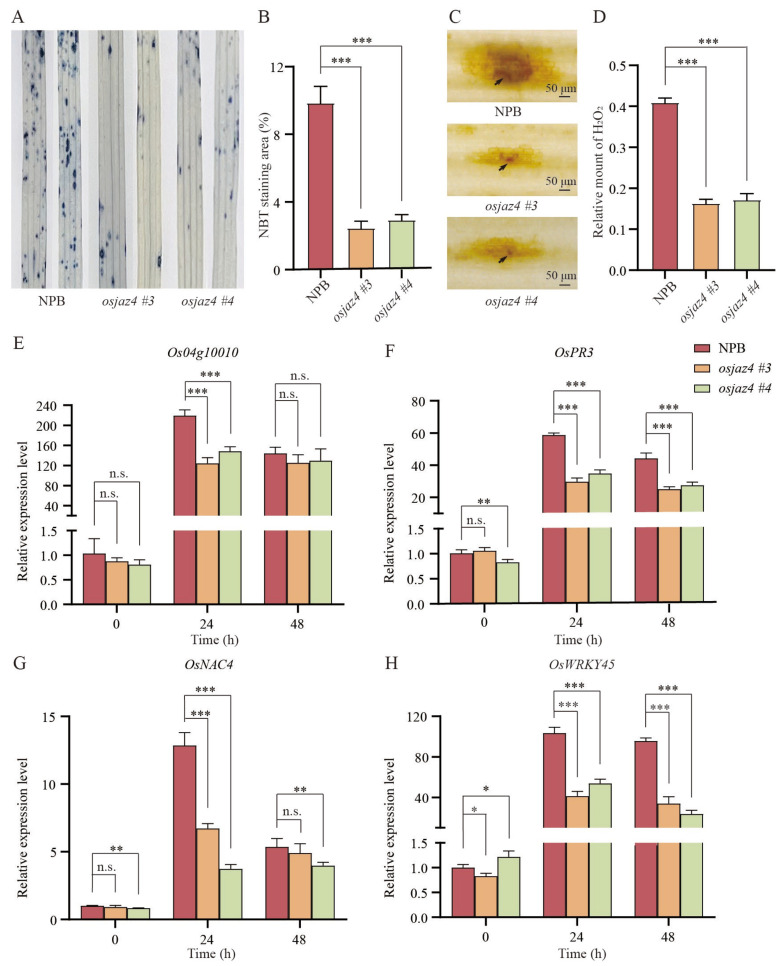
The immunity response of *osjaz4* against *M. oryzae* is weakened. (**A**) NBT staining of the infection in *osjaz4* and NPB plants inoculated with *M. oryzae* at 24 h. The blue part indicates the existence of O^2−^. (**B**) The percentage of NBT staining areas. The percentage of staining area per leaf was scored via ImageJ 1.52i with the formula: staining area (%) = pixels of the blue part/pixels of leaf × 100%. Error bars indicate SD (n = 5 leaves). Data were statistically analyzed by one-way ANOVA followed by Dunnett’s multiple comparisons test. (**C**) DAB staining of the infection sites in *osjaz4* and NPB plants at 48 h. The reddish-brown color of leaves was observed as H_2_O_2_ generation. The arrows indicate the infection structures of appressoria. Scale bar = 50 μm. (**D**) The relative amount of H_2_O_2_. The relative amount of H_2_O_2_ was calculated on the basis of the pixels of images via ImageJ 1.52i with the following formula: H_2_O_2_ area per rectangle = pixels of H_2_O_2_ area per mycelial invasion site/pixels of the rectangle. Error bars indicate SD (n = 5 leaves). Data were statistically analyzed by one-way ANOVA followed by Dunnett’s multiple comparisons test. (**E**–**H**) *M. oryzae*-induced expression of defense-related genes in 2-week-old seedlings of NPB and *osjaz4*. The relative expression levels were normalized to the level of the NPB plants at 0 h. Error bars indicate SD (n = 3). Discovery was determined by a *t*-test using the two-stage linear step-up procedure of Benjamini, Krieger, and Yekutieli, with Q = 1%. Asterisks indicate significant differences (n.s., not significant, * *p* ≤ 0.05, ** *p* ≤ 0.01, *** *p* ≤ 0.001). Three independent biological replicates with the three technical repeats were tested, each yielding similar results.

To ascertain whether basal resistance intensifies blast development in *osjaz4*, we examined several hallmark immune responses, such as the accumulation of reactive oxygen species (ROS) and the expression of defense-related genes. ROS bursts are characteristic features of pattern-triggered immunity (PTI) and effector-triggered immunity (ETI) pathways [36,37]; hence, we evaluated the accumulation of O^2−^ and H_2_O_2_ due to the infection of *M. oryzae* via nitro blue tetrazolium (NBT) staining and 3,3’-diaminobenzidine (DAB) staining, respectively. After NBT staining, the leaf staining area on the *osjaz4* leaves was smaller compared to that of NPB, indicating diminished O^2-^ production in *osjaz4* compared to the WT plants (Figure 3A,B). DAB staining revealed that *osjaz4* produced much smaller amounts of H_2_O_2_ than the WT plants in response to rice blast (Figure 3C,D).

Following pathogen assault, a cascade of defense-related genes is activated in plants, including the dehydrogenase gene *OsO4g10010* [38], the SA signaling marker gene *OsWRKY45* [39], the transcription factor gene *OsNAC4* [40], and the chitinase gene *OsPR3* [41]. Next, we examined the expression of these defense-related genes in the transgenic lines and WT plants at 0, 24, and 48 h post-inoculation with rice blast via qRT-PCR. The infection with *M. oryzae* strain 70-15 induced the expression of four defense-related genes in both the control and *osjaz4*. However, the expression of four defense-related genes at 24 and 48 h post-inoculation in *osjaz4* was markedly lower than that in the control (Figure 3E–H). Utilizing rice Ubiquitin as the internal reference gene, the data consistently revealed a concordant expression trend (Figure S4). These results implied that the mutation of *OsJAZ4* decreases both ROS bursts and the expression of defense-related genes triggered by *M. oryzae*.

### 2.4. The Content of JA in osjaz4 Is Lower Than That in Wild Type

**Figure 4:**
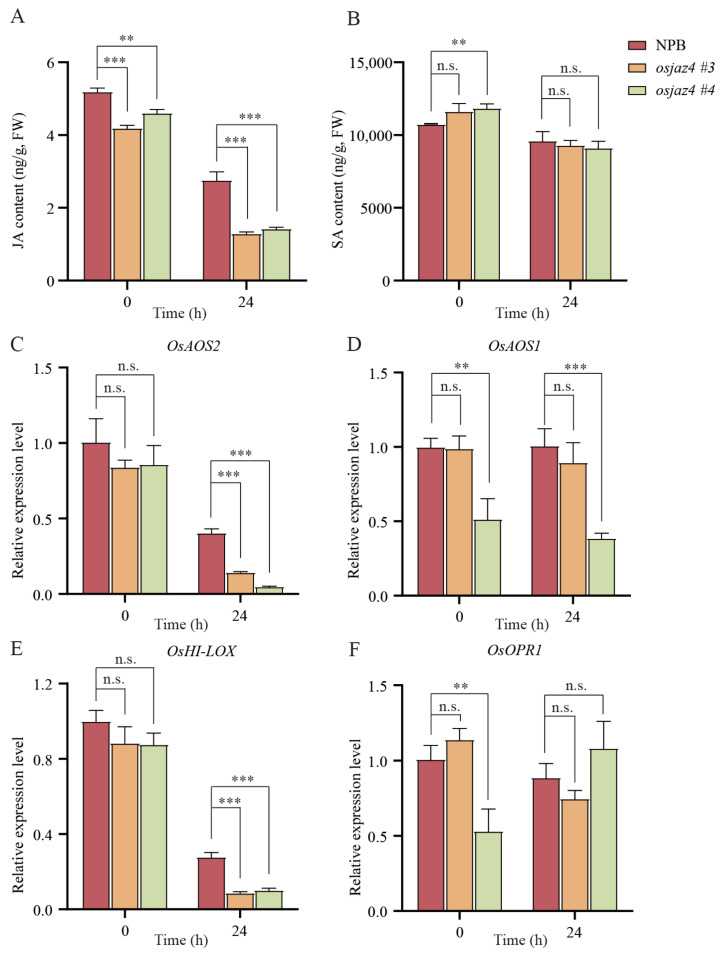
The content of JA in *osjaz4* is lower than that in NPB. (**A**,**B**) The content of endogenous SA and JA in *osjaz4* and NPB upon *M. oryzae* infection at 0 and 24 h. (**C**–**F**) Expression of genes involved in JA biosynthesis in *osjaz4* and NPB inoculated with *M. oryzae* isolate 70-15 at 0 and 24 h. The relative expression levels were normalized to the level of NPB plants at 0 h. Error bars indicate SD (n = 3). Discovery was determined by a *t*-test using the two-stage linear step-up procedure of Benjamini, Krieger, and Yekutieli, with Q = 1%. Asterisks indicate significant differences (n.s., not significant, ** *p* ≤ 0.01, *** *p* ≤ 0.001). Three independent biological replicates with three technical repeats were tested, each yielding similar results.

JAZ protein is a pivotal element of the JA signaling pathway [42] and participates in the SA signaling pathway [26]. SA and JA exert crucial roles in modulating plant defense responses [43,44]. To explore the impact of the *OsJAZ4* gene knockout on the JA and SA pathways, we assessed the endogenous JA and SA levels in the 2-week-old rice seedlings inoculated with *M. oryzae* isolate 70-15 at 0 and 24 h. The endogenous JA content in both *osjaz4* and the WT plants was low, indicating a decrease in response to *M. oryzae* infection (Figure 4A). Specifically, the JA content in the *osjaz4* plants was significantly lower than that in NPB after rice blast infection. However, the content of endogenous SA in *osjaz4* and the WT plants was high, and no significant difference was detected in the SA content between *osjaz4* and NPB after 24 h of *M. oryzae* inoculation (Figure 4B). According to these results, reduced JA accumulation in the *osjaz4* lines under normal or *M. oryzae*-inoculation conditions suggests a potential positive role of *OsJAZ4* in the regulation of the JA content.

In addition, we investigated the expression of the JA biosynthesis genes: *OsHI-LOX*, *OsOPR1*, *OsAOS1*, and *OsAOS2* [45]. Following inoculation with rice blast fungus, the expression of *OsHI-LOX* and *OsAOS2* was downregulated and significantly lower in the *osjaz4* lines than that in the WT plants. Conversely, the expression of *OsAOS1* and *OsOPR1* was subtly modulated after inoculation, with no noticeable discrepancy between *osjaz4* and NPB (Figure 4C–F). This is consistent with the results obtained using rice Ubiquitin as the internal reference gene (Figure S5). These findings implied that *OsJAZ4* has an impact on the expression of JA biosynthesis genes: *OsHI-LOX* and *OsAOS2*.

### 2.5. OsJAZ4 Function in Rice Yield Traits

**Figure 5:**
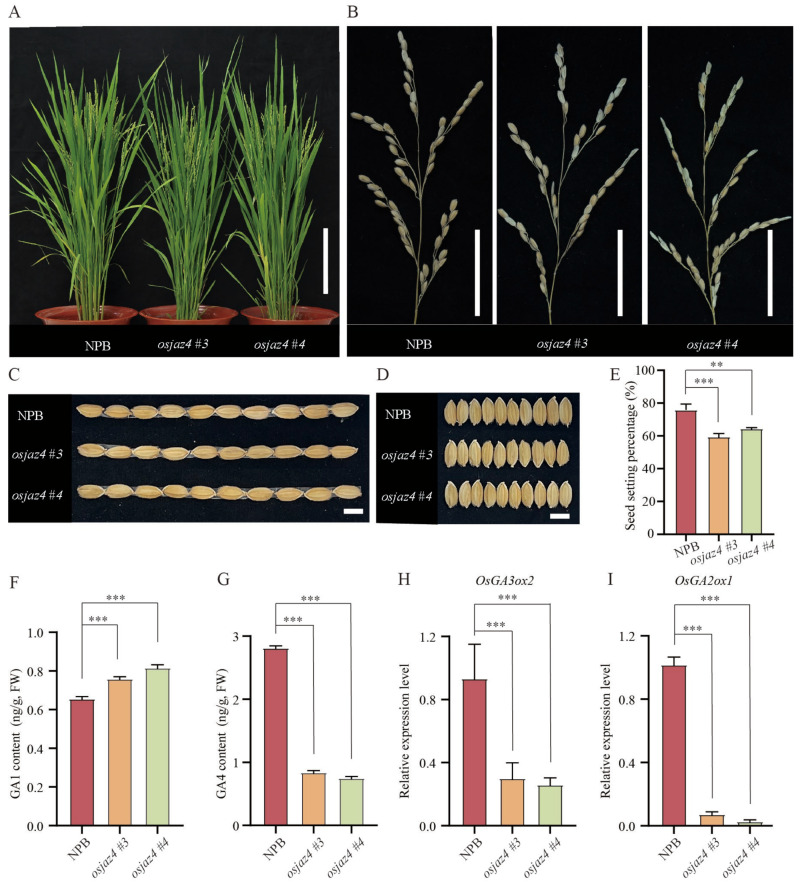
*OsJAZ4* function in rice yield traits. (**A**) Morphology of mature *osjaz4* and NPB plants in normal field conditions. Bar = 20 cm. (**B**–**D**) Panicle types, grain length, and grain width of *osjaz4* and NPB; bar = 5 cm; bar = 0.5 cm. (**E**) Percentage of seed setting *osjaz4* and NPB (n = 10). (**F**,**G**) The content of endogenous GA1 and GA4 in *osjaz4* and NPB. (**H**,**I**) qRT-PCR analysis of genes associated with GA synthesis pathway in *osjaz4* and NPB. Error bars indicate SD (n = 3). Data were statistically analyzed by one-way ANOVA followed by Dunnett’s multiple comparisons test. Asterisks indicate significant differences (** *p* ≤ 0.01, *** *p* ≤ 0.001). Three independent biological replicates were tested, each yielding similar results.

In addition to examining resistance to rice blast, we also investigated whether *OsJAZ4* influences rice growth and yield. During the mature stage, *osjaz4* did not display significant deviations in plant height and tiller number compared to NPB (Figure 5A, Figure S6A,B). The spike length was similar between *osjaz4* and the WT (Figure S6C). In addition, *osjaz4* displayed a similar panicle size but with fewer filled grains than the control (Figure 5B). Moreover, *osjaz4* exhibited a phenotype featuring a reduced seed set rate; the seed set percentage of *osjaz4 #3* and *osjaz4 #4* was 59.47% and 64.41%, respectively, while NPB had a set rate of 75.91% (Figure 5E), indicating an impact of *OsJAZ4* on the spikelet fertility in rice. Similarly, the 1000-grain weight of *osjaz4* was lower than that of the WT plants (Figure S6D). However, the length and width of the grain did not differ significantly between *osjaz4* and the WT plants (Figure 5C,D). In conclusion, these results indicated that altered *OsJAZ4* expression can cause defects in the seed setting rate, but no significant effects were observed on the plant height, tiller number, and grain size.

GA1 and GA4 are the key bioactive GAs governing rice growth [46]. Furthermore, SYL3-k increases the style length and yield of F1 seeds, boosting the endogenous GA4 content in rice pistils [47]. And GA acts through JA to promote stamen filament growth in *Arabidopsis* [48]. To comprehend the cause of the seed setting rate defect in normal field conditions, we estimated the GA1, GA4, IAA, and Zeatin levels in NPB and *osjaz4*. Subsequently, the GA4 content in *osjaz4 #3* and *osjaz4 #4* was 0.84 ng/g and 0.75 ng/g, respectively, which was significantly lower than that in the NPB plants (2.81 ng/g) (Figure 5G). However, the content of endogenous GA1 in *osjaz4 #3* and *osjaz4 #4* was 15.74% and 24.37% higher than that in NPB, respectively (Figure 5F), and the IAA and Zeatin content in *osjaz4* was slightly higher (less than 15%) than that in NPB (Figure S7A,B). Therefore, we speculated that the low seed setting rate of *osjaz4* may be related to the significant reduction in the GA4 content.

To elucidate the mechanism of *OsJAZ4’s* influence on the GA4 content, we investigated the expression levels of GA biosynthesis genes in NPB and *osjaz4* using qRT-PCR. Using *OsActin* and rice Ubiquitin as the internal reference genes, consistent results were obtained from both. The results showed that the expression level of *OsGA2ox1* and *OsGA3ox2* in *osjaz4* was significantly lower than that in NPB (Figure 5H,I), but that of the other GA biosynthesis genes, *OsCPS1*, *OsKS1*, *OsKO1*, *OsKAO*, *OsGA2ox2*, and *OsGA3ox1,* did not differ significantly (Figure S8). These findings suggested that the reduced GA4 content in *osjaz4* plants is attributed to the diminished expression of *OsGA2ox1* and *OsGA3ox2*.

## 3. Discussion

A high yield and disease resistance are the two main goals of rice breeding. Thus, studying the mechanism of the high-yield and disease-resistance balance of crops is crucial for rice breeding. In the present study, we demonstrated that *OsJAZ4* fine-tunes the rice plague resistance and yield characteristics. The *OsJAZ4* gene knockout weakens rice resistance to rice plague and reduces the fruiting rate, indicating a positive role of the *OsJAZ4* gene in both the rice yield and rice plague resistance, which provides an experimental basis for the cultivation of high-yield disease-resistant varieties.

When attacked by rice blast fungus, the ROS content and the expression of resistance-related genes in *osjaz4* are significantly lower than that in the WT (Figure 2). ROS signaling modulates a broad range of biological processes in cell expansion, development, and responses to biotic and abiotic stimuli [49,50,51]. Previous studies have shown that ROS enhances the hypersensitivity reaction of plants and can also be used as a signal for defense reactions, causing the upregulation of resistance-related genes or interaction with other signal molecules to participate in the disease-resistance process of plants [52]. Strikingly, the interaction between rice and rice blast produces ROS in rice cells and improves the resistance to rice blast [53]. Based on these conclusions, we speculated that the susceptibility to rice plague in *osjaz4* is mainly due to the weakening of ROS outbreak and the resistance-related upstream genes.

In this study, we found the mutation of *OsJAZ4* weakens the resistance to rice blast (Figure 2) and reduces the JA content (Figure 4A). Previous studies have shown that JA in rice regulates the immune response to (semi) biotrophic pathogens [2,31]. Another study [54] revealed that the loss-of-function of the Sekiguchi lesion (SL) in the rice cultivar Minghui 86 increases the content of JA and enhances resistance to *Pyricularia oryzae*. Thus, we speculated that the reduction in the rice blast resistance in *osjaz4* is related to the decreased JA content. Nonetheless, the specific mechanism of the JA-regulated disease resistance of rice blast in rice needs to be clarified further. Since the resistance to biotrophic pathogens is regulated by SA-dependent signaling pathways [55], we also considered the impact of the SA content. However, no significant difference was detected in the SA content after 24 h post-rice blast infection (Figure 4B), and the SA content is not essential for inducing resistance because rice plants usually accumulate high levels of SA during normal growth [43,56].

Our results show that the content of endogenous GA4 was dramatically reduced in *osjaz4* (Figure 5G), and the percentage of seed set was dramatically decreased compared to the WT plants (Figure 5E). These results showed that *OsJAZ4* regulates the content of GA4 in rice. GA is a critical regulating and development hormone of the fruit tree. Enhancing the synthesis of GA4 can promote pear and single-fruit development [57]. Some studies have shown that enhanced endogenous GA4 content in *Oryza sativa* L. pistils increases the style length and yield of F1 seeds in rice [47]. Therefore, we speculated that *OsJAZ4* controls the GA4 content of rice to affect the seed setting rate, and the diminished expression of GA synthetic genes, *OsGA2ox1* and *OsGA3ox2*, might be ascribed to the reduced GA4 content in *osjaz4*. Nonetheless, the content of GA1 in *osjaz4* is only increased slightly (Figure 5).

The consensus proposes that improved plant disease resistance is accompanied by decreased growth and yield [58]. GA and JA are critical plant hormones that coordinate and control the growth and defense of plants through the interaction between DELLAs and JAZs [26,59]. Our data demonstrated the impact of *OsJAZ4* on JA and GA biosynthesis; whether *OsJAZ4* is related to the crosstalk between the JA and GA pathways remains to be determined. DELLA proteins are repressors of the GA pathway and are degraded to promote plant growth [60]. These molecules also impede the JAZ–MYC interaction that mediates the antagonism of GA on JA-mediated defense. Moreover, JAZs interfere with the DELLA–PIF interaction, mediating the inhibitory effect of JA on GA-mediated growth [61]. JA and GA also synergistically regulate trichome initiation, sesquiterpene biosynthesis, and stamen development in *Arabidopsis* [62]. GA regulates JA biosynthesis via DELLAs and controls the expression of *MYB21*, *MYB24*, and *MYB57* through jasmonate to promote stamen filament [48]. Thus, it could be speculated that JA and GA have a collaborative mediating mechanism in rice. OsJAZ8 and OsJAZ9 interact with the rice DELLA protein SLE-NDER RICE 1 (SLR1); OsJAZ9 also interacts with the SLR1-LIKE protein (SLRL2). This interaction regulates the response to GA and JA signals [63]. Therefore, whether OsJAZ4 interacts with the rice DELLA protein is yet to be explored and the role of *OsJAZ4* in the crosstalk between the JA and GA pathways is to be determined. Based on the comprehensive analysis of the endogenous JA and GA content, disease resistance, and yield phenotype, the current data demonstrated a decreased content of JA and GA4 in *osjaz4*, and the penalty of yield losses accompanied a decreased broad-spectrum disease resistance in the *osjaz4* mutant. Therefore, we speculated that *OsJAZ4* positively regulates the rice blast resistance and yield by positively regulating the JA and GA content.

## 4. Materials and Methods

### 4.1. Sequence Conservation Analysis and Phylogenetic Tree Construction

Protein sequence data of 15 OsJAZ family members were retrieved from the China Rice Data Center (https://ricedata.cn/gene/) accessed on 6 February 2022 and aligned using ClustalW in MEGA 11.0.11 with default settings. Then, the phylogenetic tree of OsJAZs was constructed using MEGA 11.0.11 and neighbor-joining (NJ) method with the following analysis parameters: none test of phylogeny, Poisson model, uniform rates among sites, and pairwise deletion for gaps data treatment.

### 4.2. Plant Materials and Growth Conditions

NPB used for transgenic analysis was preserved for the long-term by the laboratory. The *osjaz4* mutants were generated in NPB background using a CRISPR/Cas9 system. Based on the coding sequence of *OsJAZ4* (*LOC_Os09g23660*), the guide RNA (gRNAs) targets in *OsJAZ4* and primers were selected via the E-CRISP Design Tool (http://www.e-crisp.org) accessed on 12 April 2022. The 20 bp target sequences were inserted into the Cas9 expression backbone pYLCRISPR/Cas9Pubi-H vector. The sequence of the resulting vector was verified and introduced into *Agrobacterium tumefaciens* EHA105 to infect the NPB. The transgenic rice lines were subsequently confirmed by sequencing the PCR products, two mutant lines *osjaz4 #3* and *osjaz4 #4*, used for subsequent phenotypic assays.

The WT and transgenic plants were grown in a greenhouse at 26 °C and 12 h photoperiod under normal field conditions from May–September in Taohua village, Changsha, China (28°11′ N, 112°58′ E). The plant height and tiller number were measured from 15 plants in the paddy fields at full maturity. The spike length, seed setting percentage, and 1000-grain weight were measured manually; the 1000-grain weight was measured after the grains were dried in a 37 °C oven for 4 d. The samples’ normal distribution was assessed using GraphPad Prism 8.0 software via the Shapiro–Wilk test. Data were statistically analyzed by one-way ANOVA followed by Dunnett’s multiple comparisons test with adjusted *p* values using GraphPad Prism 8.0 software.

### 4.3. Fungal Strains and Culture Conditions

The 70-15 is an *M. oryzae* isolate maintained in our laboratory. Guy11 was generously provided by Dr. Xuewei Chen (Sichuan Agricultural University). HNB52 and HNB119 were collected from Hunan Province; HNB52 strain belongs to the ZC16 physiological race, and HNB119 belongs to the ZB13 physiological race [64]. All strains were cultured on agar plates containing complete medium (CM) at 25 °C under 12 h/12 h light/dark cycles.

### 4.4. Pathogenicity Assays

Conidia were collected after 7–10 d of culture, harvested with 0.025% Tween-20, and inoculated at the wound sites at the concentration of 1.5 × 10^5^ conidia/mL spores, as described previously [65]. Moreover, the leaves detached from 3-week-old seedlings were teased with a needle for wound inoculation. For spray inoculation, conidia collected with 5 g/L gelatin were inoculated onto 2-week-old seedlings at the concentration of 1.5 × 10^5^ conidia/mL spores. Subsequently, leaves or seedlings were maintained in a moist chamber at 28 °C for 24 h in the dark, followed by a 12 h light/12 h dark cycle for 6 d. The percentage of lesion areas per leaf and the length of the lesion were scored using ImageJ 1.52i. The normal distribution of the samples was examined using GraphPad Prism 8.0 software via the Shapiro–Wilk test. Statistical analysis was performed using GraphPad Prism 8.0 software, involving one-way ANOVA followed by Dunnett’s multiple comparisons test, with adjusted *p* values.

### 4.5. O^2−^ and H_2_O_2_ Accumulation

NBT staining can locate the site of O^2−^ production and was used to examine O^2−^ accumulation in fresh plant tissue. The leaves of 2-week-old rice seedlings were soaked in NBT solution (0.1%, pH 7.8; Coolaber, Nanjing, China) after 24 h of spray inoculation with *M. oryzae* isolate 70-15 (1.5 × 10^5^/mL spores). Then, a negative pressure vacuum was applied at −0.1 MPa for 30 min, and the isolate was placed at room temperature (25 °C) in the dark for 12 h. The NBT-stained leaves were cleaned by boiling in 95% ethanol for 20 min, and images were captured with iPhone 13 (Apple Inc., Cupertino, CA, USA). The blue part indicates the existence of O^2−^. The percentage of staining area per leaf was scored via ImageJ 1.52i with the formula: staining area (%) = pixels of the blue part/pixels of leaf × 100%.

DAB staining was performed as described previously with some modifications [66]. Two-week-old leaves were collected 48 h after spray inoculation with *M. oryzae* isolate 70-15 and placed in DAB solution (1 mg/mL, pH 3.5; Coolaber). Samples were vacuum-infiltrated for 30 min and then incubated for 8 h at 25 °C with gentle shaking at 75 rpm. Next, the chlorophyll in the samples was removed by boiling in 95% ethanol for 20 min. The samples were observed, and images taken under Smartzoom 5 microscope (Carl Zeiss AG, Oberkochen, Germany). The reddish-brown color of leaves was observed as H_2_O_2_ generation. The relative amount of H_2_O_2_ was calculated on the basis of the pixels of images via ImageJ 1.52i with the formula: H_2_O_2_ area per rectangle = pixels of H_2_O_2_ area per mycelial invasion site/pixels of the rectangle. The samples’ normal distribution was assessed using GraphPad Prism 8.0 software via the Shapiro–Wilk test. The statistical analysis of O^2−^ and H_2_O_2_ was performed by one-way ANOVA followed by Dunnett’s multiple comparisons test with adjusted *p* values in GraphPad Prism 8.0 software.

### 4.6. RNA Extraction and qRT-PCR

Total RNA was extracted from the leaves of 2-week-old rice seedlings using TRIzol reagent according to the manufacturer’s protocol (Invitrogen, Carlsbad, CA, USA). The RNA integrity was examined via 1% agarose gel electrophoresis, and the RNA concentration and quality were determined using a NanoDrop 2000 UV–vis spectrophotometer (Thermo Fisher Scientific, Waltham, MA, USA). Reverse transcription was carried out using All-In-One 5X RT MasterMix according to the manufacturer’s protocol (Abm, Shanghai, China). qRT-PCR was conducted using the 5× qRT-PCR MasterMix (Abm) on a LightCycler 480Ⅱ (Roche, Basel, Switzerland). The parameters for amplification were as follows: initial denaturation at 94 °C for 5 min; 30 cycles of denaturation at 94 °C for 30 s, annealing at 60 °C for 30 s, and extension at 72 °C for 50 s; final extension at 72 °C for 3 min. The rice gene *OsActin* (*LOC_Os03g50885*) and rice Ubiquitin gene (*LOC_Os03g13170*) were used as an internal control. The 2^−ΔΔCT^ method was used to calculate the relative expression of the target gene. The statistical analysis of gene expression was performed in GraphPad Prism 8.0 software using one-way ANOVA followed by Dunnett’s multiple comparisons test with adjusted *p* values, or a *t*-test applying the two-stage linear step-up procedure of Benjamini, Krieger, and Yekutieli. The heatmaps were created using Evolview (https://www.evolgenius.info/evolview) accessed on 26 February 2022. The primers used are listed in Table S1. Primer sequences for some of the primers were sourced from literature references [45,67,68,69,70,71]. Table S1 presents the sequences and pertinent details for each primer.

### 4.7. Electrospray Ionization Mode (ESI)–High-Performance Liquid Chromatography (HPLC)–-Mass Spectrometry (MS)/MS Analysis of JA, SA, GA1, GA4, IAA, and Zeatin Content

A spore suspension (1.5 × 10^5^ conidia/mL) of *M. oryzae* isolate 70-15 was inoculated on the rice leaves of the NPB and jaz4 mutants at the three-leaf stage via spraying. An equivalent of 2 g of the leaves’ tissue sample was collected at 0 and 24 h post-inoculation, rapidly frozen in liquid nitrogen, and stored at −80 °C. Then, D-JA, D-SA, D-GA1, D-GA4, D-IAA, and D-Zeatin were mixed with 994 μL methanol, and the final concentration was configured to 1 μg/mL internal standard to use as mother liquor. Then, the sample was ground in liquid nitrogen, weighed accurately, and 10 mL of acetonitrile and 8 μL of internal standard mother liquor was added. The supernatant was obtained via centrifugation at 12,000× *g*, 4 °C for 5 min. A volume of 5 mL of acetyl was added to the precipitate, extracted twice, and combined with the supernatant. An appropriate amount of C18 and GCB was added to purify the impurities via centrifugation at 12,000× *g*, 4 °C for 5 min on a TG-16G table-top high-speed centrifuge (Kaidalab, Hunan, China). Then, the supernatant was dried with nitrogen (NAI, Shanghai, China), re-dissolved with 300 μL methanol, filtered through a 0.22 μm organic-phase filter membrane, and analyzed via HPLC (1260, Agilent Technologies, Santa Clara, CA, USA) and MS (6420A, Agilent Technologies). A volume of 2 μL of each sample was injected at a flow rate of 0.3 mL/min onto a Poroshell 120-SB-C18 reverse-phase column (2.1 × 150, 2.7 m) at the column temperature 30 °C. A mobile phase composed of solvent A (0.1% formic acid in methanol) and solvent B (0.05% formic acid in H_2_O) was used in a gradient mode for separation. The gradient elution mode was as follows: 0–1 min, 20% A; 1–3 min, 20–50% A; 3–9 min, 50–80% A; 9–10.5 min, 80% A; 10.5–10.6 min, 80–20% A; 10.6–13.6 min, 20%. An ESI, positive ion mode (+4500 V), and negative ion mode (−4000 V) were used for detection. The MS conditions were as follows: atomization gas pressure 65 psi; auxiliary gas pressure 70 psi; atomization temperature 400 °C; multiple reaction monitoring type of scan. The data were statistically analyzed in GraphPad Prism 8.0 software using one-way ANOVA followed by Dunnett’s multiple comparisons test with adjusted *p* values, or a *t*-test employing the two-stage linear step-up procedure of Benjamini, Krieger, and Yekutieli.

## 5. Conclusions

The mutation of *OsJAZ4* weakens rice’s resistance to *M. oryzae* and reduces the seed setting rate, indicating a positive role of the *OsJAZ4* gene in both the rice yield and rice blast resistance. As the JA and GA4 content in the *osjaz4* mutants was significantly lower than that in the wild type, we speculated that *OsJAZ4* tailors the rice blast resistance and yield by regulating the content of JA and GA4. Thus, our results highlight potential genetic tools for the simultaneous improvement in the blast resistance and yield traits, indicating a step forward in resolving growth-defense trade-offs in crops.

## Data Availability

Data is contained within the article.

## Supplementary Materials

The following supporting information can be downloaded at: https://www.mdpi.com/article/10.3390/plants13030348/s1, Figure S1: the expression patterns of the *OsJAZs* in response to *M. oryzae* infection, normalized to Ubiquitin levels; Figure S2: identification of *osjaz4* mutant; Figure S3: analysis of the resistance of *OsJAZ4* transgenic lines to different *M. oryzae* strains; Figure S4: *M. oryzae*-induced defense-related gene expression in 2-week-old NPB and *osjaz4* seedlings, with Ubiquitin as the internal control; Figure S5: expression of genes involved in JA biosynthesis in *osjaz4* and NPB, with Ubiquitin as the internal control; Figure S6: plant height, tiller number, spike length, and 1000-grain weight of *osjaz4* and NPB; Figure S7: IAA and Zeatin content of *osjaz4* and NPB; Figure S8: expression of genes involved in GA biosynthesis in *osjaz4* and NPB; Table S1: list of primers used in this study.
