# Supplementary material for: OsJAZ4 Fine-Tunes Rice Blast Resistance and Yield Traits
Source: Plants (Basel). 2024 Jan 24;13(3):348. doi: 10.3390/plants13030348 (PMC10857531; doi:10.3390/plants13030348)
Supplement: Supplementary file 1 [file plants-13-00348-s001.zip › Figure S1-S8.pdf]

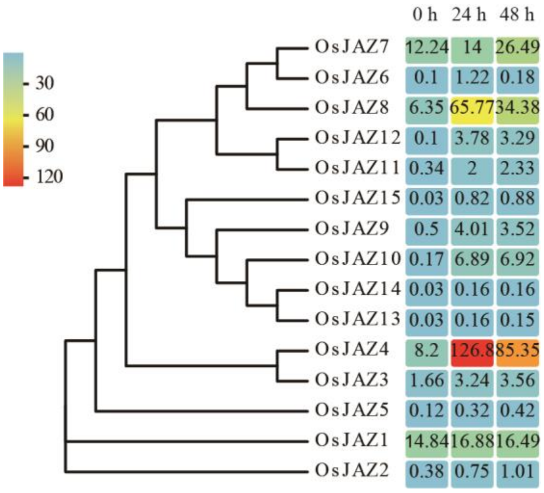

FigureS1. The expression patterns of the *OsJAZs* in response to *M. oryzae* infection, normalized to Ubiquitin levels. The expression levels from low to high are indicated by a change in the color from blue to red. Three independent biological replicates with the three technical repeats were tested, each yielding similar results.

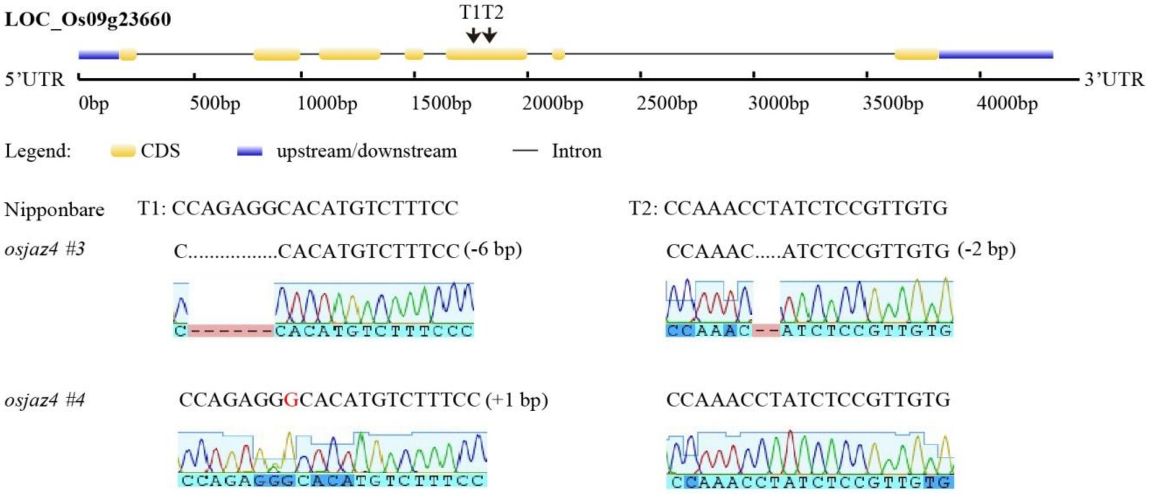

FigureS2. Identification of *osjaz4* mutant. Sequence alignment of the gRNAs target region shows deleted or inserted bases in the *osjaz4* mutant; bases inserted are shown in red.

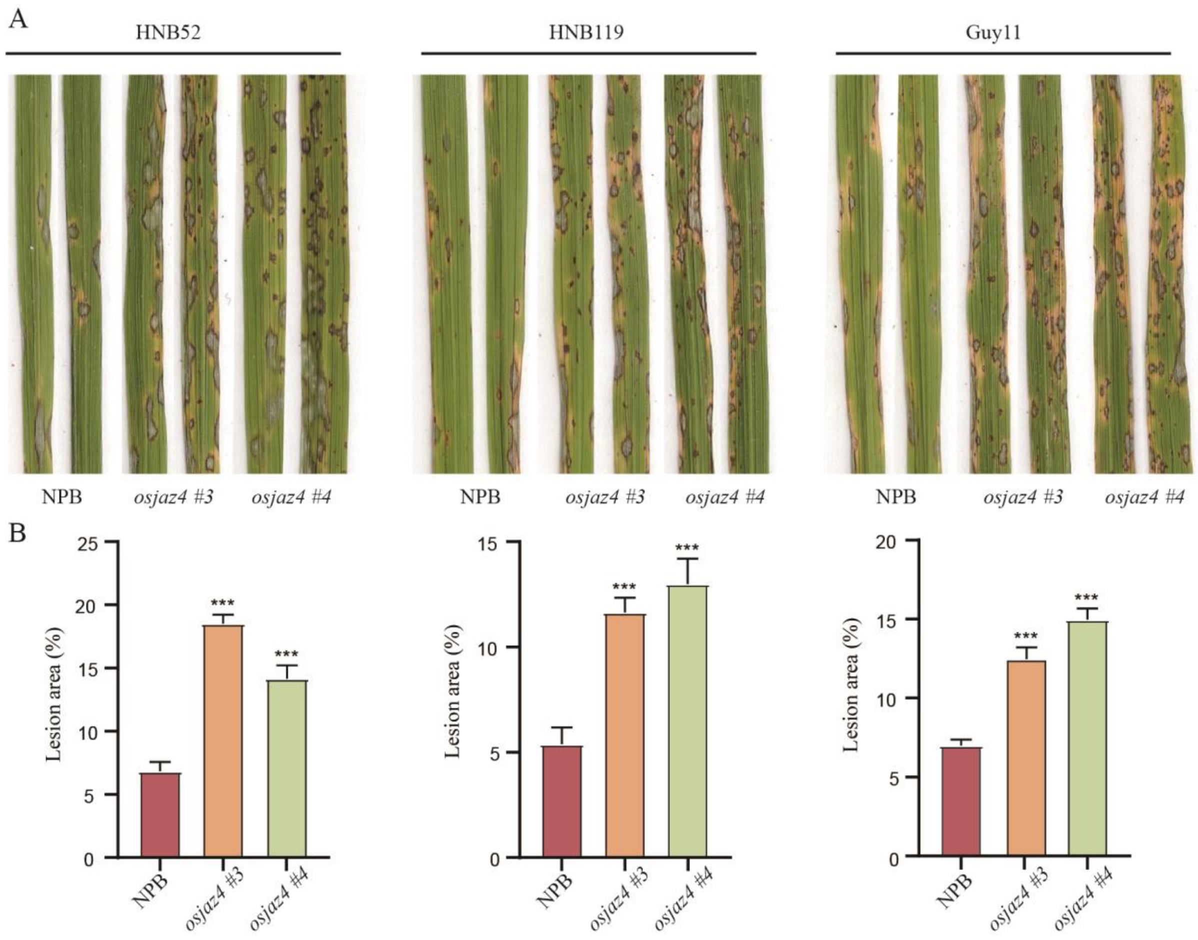

FigureS3. Analysis of the resistance of *OsJAZ4* transgenic lines to different *M. oryzae* strains. (A) Phenotypes of 2-week-old seedlings of NPB, *osjaz4* spray-inoculated with *M. oryzae* isolates HNB52, HNB119, and Guy11 at a concentration of 1.5×10^5 conidia/mL at 6 dpi. (B) Analysis of the percentage of lesion areas of the inoculated leaves (n=5). The percentage of lesion areas was scored via image analysis with ImageJ software. Error bars indicate SD. Data are statistically analyzed by one-way ANOVA followed by Dunnett's multiple comparisons test. The asterisks indicate significant differences (*** $P \leq 0.001$). Three independent biological replicates with three technical repeats were assessed, each yielding similar results.

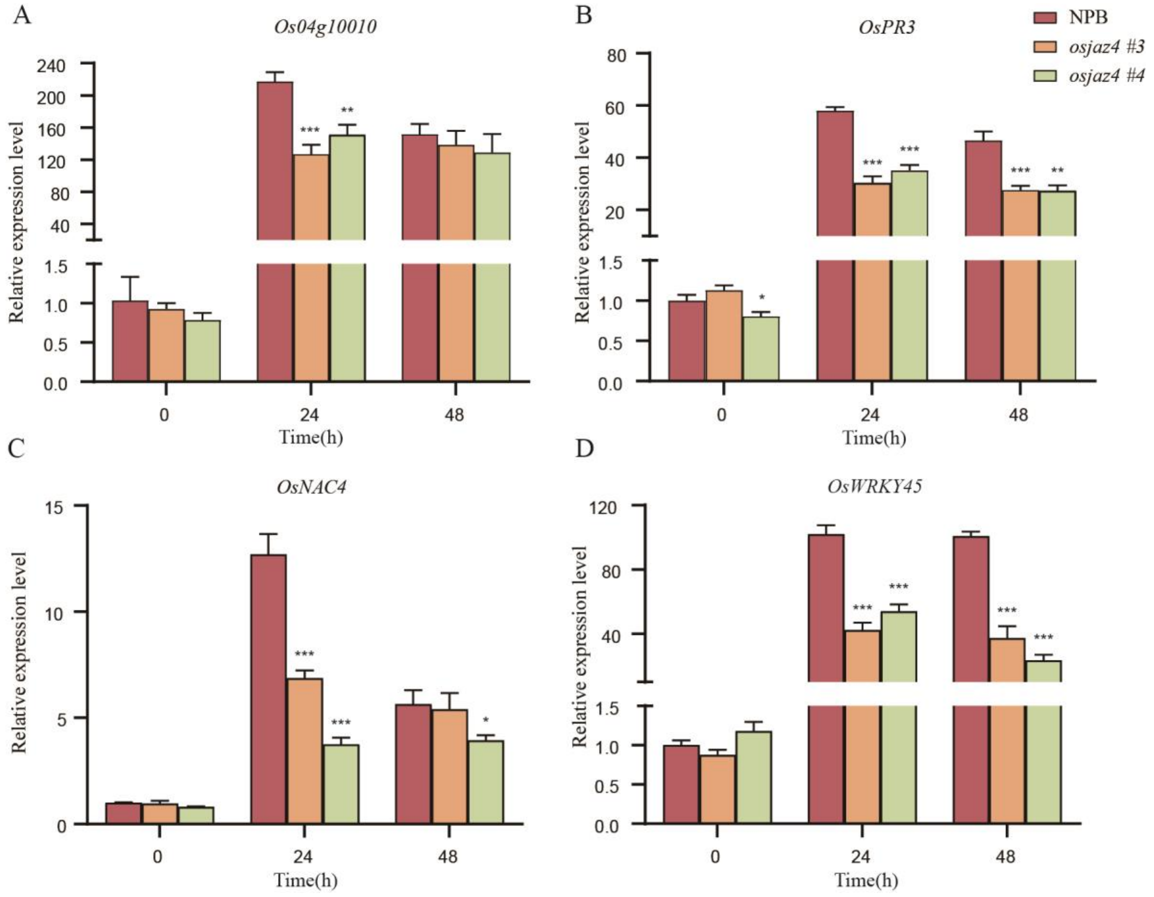

FigureS4. *M. oryzae*-induced defense-related gene expression in 2-week-old NPB and *osjaz4* seedlings, with Ubiquitin as internal control. The relative expression levels were normalized to the level of the NPB plants at 0 h. Error bars indicate SD (n=3). Discovery was determined by a t-test using the two-stage linear step-up procedure of Benjamini, Krieger, and Yekutieli, with Q=1%. Asterisks indicate significant differences (* P≤0.05, ** P≤0.01, *** P≤0.001). Three independent biological replicates with the three technical repeats were tested, each yielding similar results.

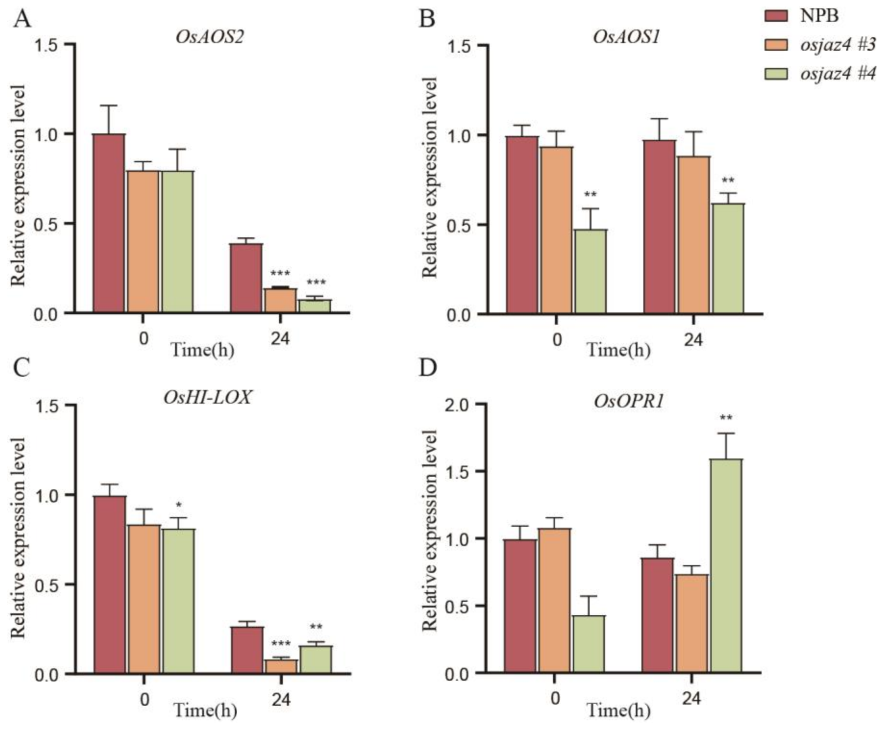

FigureS5. Expression of genes involved in JA biosynthesis in *osjaz4* and NPB inoculated with *M. oryzae* isolate 70-15 at 0 and 24 h, with Ubiquitin as internal control. The relative expression levels were normalized to the level of NPB plants at 0 h. Error bars indicate SD (n=3). Discovery was determined by a t-test using the two-stage linear step-up procedure of Benjamini, Krieger, and Yekutieli, with Q=1%. Asterisks indicate significant differences (* P≤0.05, ** P≤0.01, *** P≤0.001). Three independent biological replicates were tested, each yielding similar results

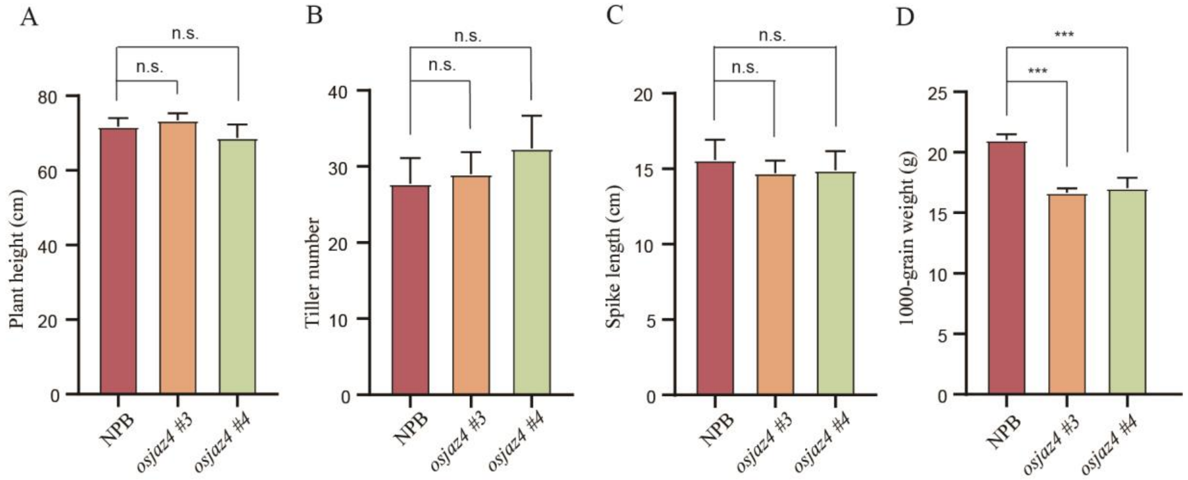

FigureS6. Plant height, tiller number, spike length, and 1,000-grain weight of *osjaz4* and NPB. Plant height, tiller number, and spike length were measured in the paddy fields at full maturity (n=15). 1,000-grain weight was measured after grains were dried in a 37 °C oven for 4 dpi (n=5). Error bars indicate SD. Data are statistically analyzed by one-way ANOVA followed by Dunnett's multiple comparisons test. The asterisks indicate significant differences (*** P≤0.001). Three independent biological replicates with three technical repeats were assessed, each yielding similar results.

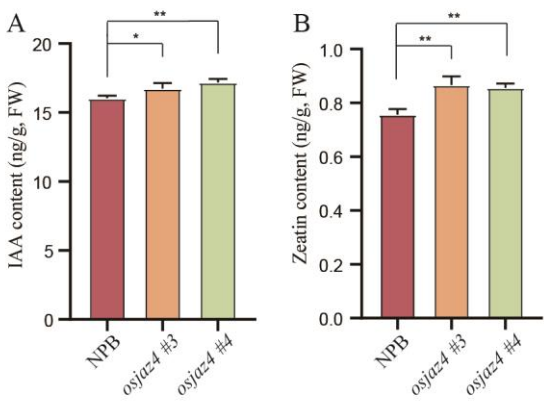

FigureS7. IAA and Zeatin content of *osjaz4* and NPB. Error bars indicate SD (n=3). Data are statistically analyzed by one-way ANOVA followed by Dunnett's multiple comparisons test. The asterisks indicate significant differences (* P≤0.05, ** P≤0.01). Three independent biological replicates with three technical repeats were assessed, each yielding similar results.

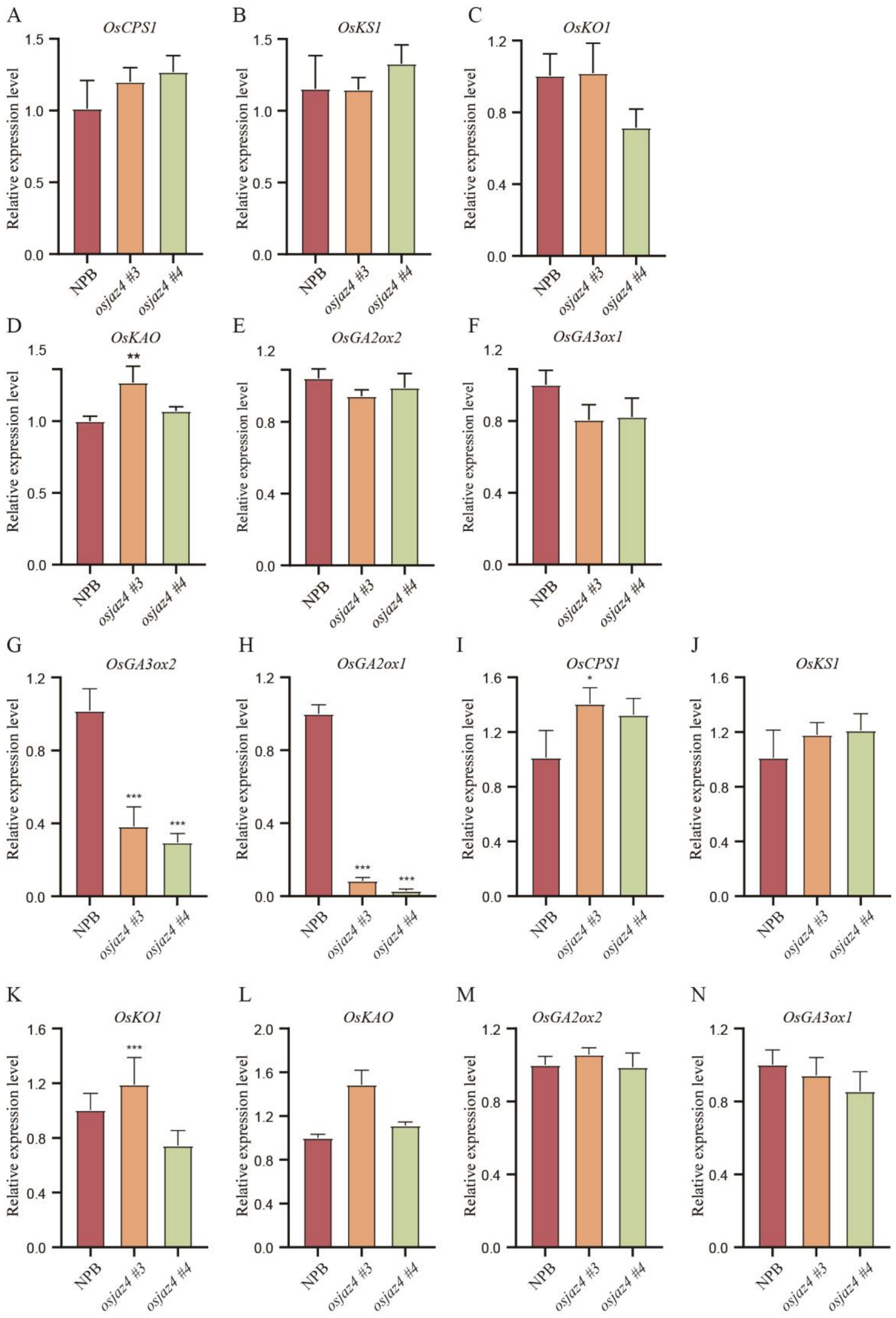

FigureS8. Expression of genes involved in GA biosynthesis in *osjaz4* and NPB. (A-F) qRT-PCR analysis of genes associated with GA synthesis pathway in *osjaz4* and NPB, with *OsActin* as internal control; (G-N) qRT-PCR analysis of genes associated with GA synthesis pathway in *osjaz4* and NPB, with Ubiquitin as internal control. Error bars indicate SD (n=3). Data are statistically analyzed by one-way ANOVA followed by Dunnett's multiple comparisons test.

Asterisks indicate significant differences (* $P \leq 0.05$, ** $P \leq 0.01$, *** $P \leq 0.001$). Three independent biological replicates were tested, each yielding similar results.
