# Supplementary material for: OsJAZ4 Fine-Tunes Rice Blast Resistance and Yield Traits
Source: Plants (Basel). 2024 Jan 24;13(3):348. doi: 10.3390/plants13030348 (PMC10857531; doi:10.3390/plants13030348)
Supplement: Supplementary file 1 [file plants-13-00348-s001.zip › Table S1.pdf]

Table S1. List of primers used in this study

Primer Name	Primers(5'-3')	Source
**For CRISPR/Cas9**		
OsJAZ4-Cas9-F	ATGTATTCAACAACGTCTCGC	This paper
OsJAZ4-Cas9-R	AATAGGCATACCTCTCGGC	This paper
**qRT-PCR for gene expression**		
OsJAZ1-RT-F	GCGTTCATTGTTTAGATGGTGT	This paper
OsJAZ1-RT-R	GCACATAAGGAACAGTACAACG	This paper
OsJAZ2-RT-F	CGAGGACGATCATATCCATGG	This paper
OsJAZ2-RT-R	CTCGTCTTCCTCTTCTCGAG	This paper
OsJAZ3-RT-F	CCTGGTGATCATTGTATACCGA	This paper
OsJAZ3-RT-R	TCATCATACCCTAACACGGATG	This paper
OsJAZ4-RT-F	CTACCACTGCTGCAGCTATTAT	This paper
OsJAZ4-RT-R	GTCAAATGAGCAATTGTCCCTT	This paper
OsJAZ5-RT-F	CAAGTCTTTTCTTGCGAAGGAA	This paper
OsJAZ5-RT-R	CGATCGATCAACACTAAACAGG	This paper
OsJAZ6-RT-F	TGACTCGAGTGATTCATGAACA	This paper
OsJAZ6-RT-R	CGTAGAAGATGGTTAATTGGCG	This paper
OsJAZ7-RT-F	TGCTTCTGATATGCCTATTGCT	This paper
OsJAZ7-RT-R	TATGGCGTCTTTGCATTAAGAC	This paper
OsJAZ8-RT-F	ATGAATGCAAATGCTCCATACC	This paper
OsJAZ8-RT-R	AGATCACTTGACACTTGACAGT	This paper
OsJAZ9-RT-F	ATATGTGATCAGCGACGTACAG	This paper
OsJAZ9-RT-R	AACTAAGCACAAGCACAAAGAG	This paper
OsJAZ10-RT-F	ACTCAAAACCACCAAAACTCAC	This paper
OsJAZ10-RT-R	CATCGCCATATCAACAAACACT	This paper
OsJAZ11-RT-F	AGTACATGAAGGAGCACAGTG	This paper
OsJAZ11-RT-R	CTTCCTTTCTTGCGTGTCTTTC	This paper
OsJAZ12-RT-F	CTCGGGCTCTGAAGATTTATCT	This paper
OsJAZ12-RT-R	TTGGGCCAAAGAAATCTCAAAC	This paper
OsJAZ13-RT-F	ACACGTCAGCTTTAATCCCATA	This paper
OsJAZ13-RT-R	AGGAATAATCGTGCACTGTACA	This paper
OsJAZ14-RT-F	GTCCGTGGAGAAGAGAAGTC	This paper
OsJAZ14-RT-R	AGCTCGAATTCAGTAGAACGC	This paper
OsJAZ15-RT-F	CCATTGACCATCGTCTACGG	This paper
OsJAZ15-RT-R	CACTTTTGCTTCCTCTTTTGGA	This paper
OsActin-RT-F	TGTATGCCAGTGGTCGTACCA	[67]
OsActin-RT-R	CCAGCAAGGTCGAGACGAA	[67]
OsUbiquitin-RT-F	GCCCAAGAAGAAGATCAAGAAC	[68]

OsUbiquitin-RT-R	AGATAACAACGGAAGCATAAAAGTC	[68]
Os04g10010-RT-F	AAATGATTTGGGACCAGTCG	[69]
Os04g10010-RT-R	GATGGAATGTCCTCGCAAAC	[69]
OsNAC4-RT-F	TCCTGCCACCATTCTGAGATG	[69]
OsNAC4-RT-R	TTGCAGAATCATGCTTGCCAG	[69]
OsWRKY45-RT-F	CGGGTAAAACGATCGAAAGA	[70]
OsWRKY45-RT-R	GCTGAGACGACACATCAACAA	[70]
OsPR3-RT-F	CTTGGACTGCTACAACCAGA	This paper
OsPR3-RT-R	CATTGTGGGCATTACTGATG	This paper
OsAOS2-RT-F	CAATACGTOTACTGGTCGAATGG	[45]
OsAOS2-RT-R	AAGGTGICOTACCGGAGGAA	[45]
OsAOS1-RT-F	TCCTCCGATACGACTCCTTC	[45]
OsAOS1-RT-R	AGTGAAGAAGAAGAGGACGG	[45]
OsLOX-RT-F	GCATCCCCAACAGCACATC	[45]
OsLOX-RT-R	AATAAAGATTIGGGAGTGACATATTGG	[45]
OsOPR1-RT-F	CAAAGGTCATTGTGCTCTTTGA	This paper
OsOPR1-RT-R	CTGAAGTCGTCGATGATCTGAG	This paper
OsCPS1-RT-F	CAGCCTCTATGAAGAAAATGGC	This paper
OsCPS1-RT-R	CACTTTTGACCTTCTCGAACTG	This paper
OsKS1-RT-F	CACTTTTCCTGGTATGCTTAGC	This paper
OsKS1-RT-R	CGGAGGTGAAGAAGTCTATCAA	This paper
OsKO1-RT-F	GAGATTTCTTCCCCTACCTCAG	This paper
OsKO1-RT-R	CCACACTAGCATCATTAGTTGC	This paper
OsKAO-RT-F	CATTTTCTCCTGGGTTACAAGC	This paper
OsKAO-RT-R	AGCGAGCATATCAAGACGAATA	This paper
OsGA2ox1-RT-F	AATACCACCACTCACTCTTAGC	This paper
OsGA2ox1-RT-R	CATGCATACTTTGACACGTAGG	This paper
OsGA2ox2-RT-F	GGAGTACAAGAGGACCATGTAC	This paper
OsGA2ox2-RT-R	CTATTCATGGTCGTCATCGTCC	This paper
OsGA3ox1-RT-F	TGATTGCAATTATCCATGCCAG	This paper
OsGA3ox1-RT-R	TCCTGGTTCTACAGTGAAACTC	This paper
OsGA3ox2-RT-F	TCTCCAAGCTCATGTGGTCCGAGGGCTA	[71]
OsGA3ox1-RT-R	TGGAGCACGAAGGTGAAGAAGCCCGAGT	[71]
